# Progranulin is Neurotrophic *In Vivo* and Protects against a Mutant TDP-43 Induced Axonopathy

**DOI:** 10.1371/journal.pone.0013368

**Published:** 2010-10-13

**Authors:** Angela S. Laird, Annelies Van Hoecke, Louis De Muynck, Mieke Timmers, Ludo Van Den Bosch, Philip Van Damme, Wim Robberecht

**Affiliations:** 1 Laboratory of Neurobiology, Department of Experimental Neurology, K.U. Leuven, Leuven, Belgium; 2 Department of Neurology, K.U. Leuven, Leuven, Belgium; 3 Vesalius Research Center, VIB, Leuven, Belgium; Brigham and Women's Hospital, Harvard Medical School, United States of America

## Abstract

Mislocalization, aberrant processing and aggregation of TAR DNA-binding protein 43 (TDP-43) is found in the neurons affected by two related diseases, amyotrophic lateral sclerosis (ALS) and frontotemporal lobe dementia (FTLD). These TDP-43 abnormalities are seen when TDP-43 is mutated, such as in familial ALS, but also in FTLD, caused by null mutations in the progranulin gene. They are also found in many patients with sporadic ALS and FTLD, conditions in which only wild type TDP-43 is present. The common pathological hallmarks and symptomatic cross over between the two diseases suggest that TDP-43 and progranulin may be mechanistically linked. In this study we aimed to address this link by establishing whether overexpression of mutant TDP-43 or knock-down of progranulin in zebrafish embryos results in motor neuron phenotypes and whether human progranulin is neuroprotective against such phenotypes. Mutant TDP-43 (A315T mutation) induced a motor axonopathy characterized by short axonal outgrowth and aberrant branching, similar, but more severe, than that induced by mutant SOD1. Knockdown of the two zebrafish progranulin genes, *grna* and *grnb*, produced a substantial decrease in axonal length, with knockdown of *grna* alone producing a greater decrease in axonal length than *grnb*. Progranulin overexpression rescued the axonopathy induced by progranulin knockdown. Interestingly, progranulin also rescued the mutant TDP-43 induced axonopathy, whilst it failed to affect the mutant SOD1-induced phenotype. TDP-43 was found to be nuclear in all conditions described. The findings described here demonstrate that progranulin is neuroprotective *in vivo* and may have therapeutic potential for at least some forms of motor neuron degeneration.

## Introduction

The biological role of progranulin (PGRN) is incompletely understood. It has been reported to be involved in development, tumor growth, wound healing and inflammation, but its role in the nervous system remains to be elucidated [1], [2], [3], [4]. We have previously demonstrated that PGRN has neurotrophic effects *in vitro*, producing increased neurite lengths and neuron survival in both cortical and motor neurons [5]. Its neuroprotective effect *in vivo* is unexplored.

Null mutations in the PGRN gene are responsible for about a third of hereditary FTLD, which itself represents about 40% of all FTLD, the second most common form of dementia in patients under 65 years of age [6], [7], [8]. These mutations produce progranulin haplo-insufficiency, evident by decreased PGRN levels in the cerebrospinal fluid and serum of patients with FTLD caused by PGRN mutations [5], [9], [10]. The brains of patients with progranulin mutations are characterized by nuclear and cytoplasmic inclusions that contain TDP-43 that is aberrantly cleaved, phosphorylated and ubiquitinated [11], [12]. Similar TDP-43 containing inclusions are also seen in the majority of patients with sporadic FTLD [13].

Missense mutations in TDP-43, on the other hand, cause amyotrophic lateral sclerosis (ALS) [14], [15], [16], [17], [18], [19]. ALS is a fatal motor neuron disease that is frequently accompanied by frontal lobe dysfunction, and sometimes by full FTLD [20], [21], [22]. ALS is mostly sporadic (90%); mutations in TDP-43 explain about 5% of the hereditary forms [14]. The motor neurons of ALS patients with TDP-43 mutations contain inclusions with abnormally cleaved, phosphorylated and ubiquitinated TDP-43, similar to those described for FTLD caused by progranulin mutations [11]. Importantly, similar inclusions are also seen in sporadic ALS patients, but not in patients with mutant SOD1-associated ALS (which accounts for about 20% of familial ALS patients)[23], [24].

The pathological and genetic links between FTLD and ALS suggest an interaction between the molecular pathways through which progranulin and TDP-43 act in the process of neurodegeneration. To study this interaction, we aimed to investigate the effect of progranulin knock down or overexpression of wild type and mutant TDP-43 on motor neuron outgrowth in the zebrafish. To investigate the role of PGRN *in vivo* we first examined the effect of knocking down zebrafish PGRN protein using morpholinos targeted to the *grna* and *grnb* genes, two fish orthologues of the human gene. Both ATG and 5′UTR morpholinos were used to exclude off target effects, and 5-base pair mismatch morpholinos were used as controls. We also aimed to confirm the effect of mutant TDP-43 mRNA expression on motor axon outgrowth and to test whether PGRN overexpression is protective against the axonopathies induced by mutant TDP-43 and SOD1.

## Results

### Knockdown of zebrafish PGRN leads to a motor axonopathy

**Figure 1:**
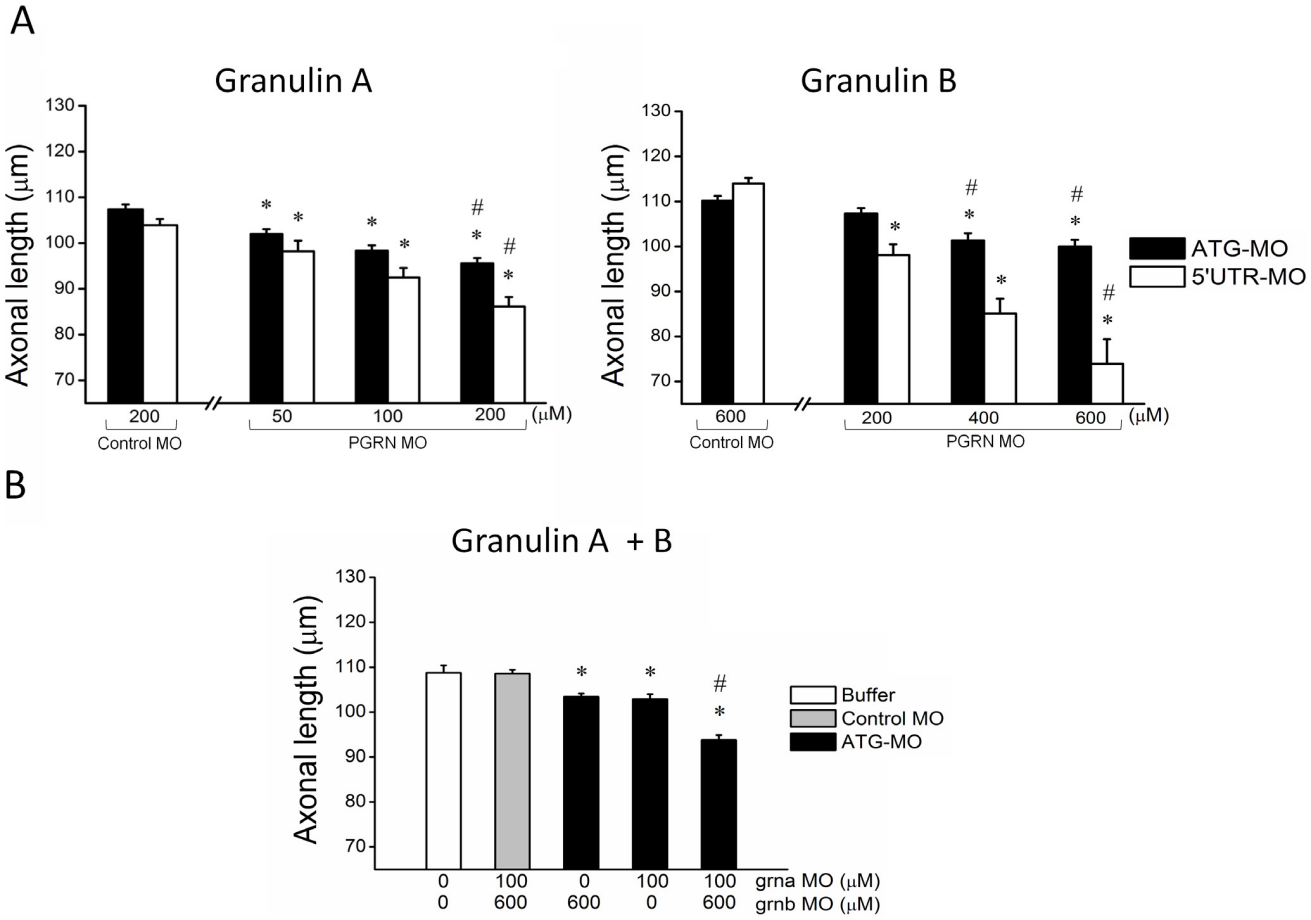
PGRN knockdown results in reduced motor axon outgrowth. A) Knockdown of *grna*, by morpholino targeted to both the start codon (ATG) and 5′UTR region of zebrafish PGRN sequence, produced a dose dependent decrease in axonal length compared to mismatch Control MO injected embryos.∧Significantly different from 200 µM Control MO, p<0.036; # significantly different from 50 µM MO, p<0.001; grna CO-MO (ATG): n = 41; grna CO-MO (UTR): n = 20; grna ATG-MO, 50 µM: n = 40, 100 µM: n = 40, 200 µM: n = 41; grna 5′UTR-MO, 50 µM: n = 27, 100 µM: n = 28, 200 µM: n = 14; Knockdown of *grnb* produced a similar, but more subtle, axonal shortening. * Significantly different from 600 µM Control MO, p<0.038; # significantly different from 200 µM MO, p<0.05; grnb CO-MO (ATG): n = 27; grnb CO-MO (UTR): n = 10; grnb ATG-MO, 200 µM: n = 40, 400 µM: n = 36, 600 µM: n = 41; grnb 5′UTR-MO, 200 µM: n = 9, 400 µM: n = 12, 600 µM: n = 12; B) The two MO used simultaneously had a cumulative effect; * significantly different from Control MO a + b, p<0.002; # significantly different from all other groups p<0.0001. Buffer injected: n = 20, CO-MO (A + B): n = 34, grnb MO: n = 36, grna MO: n = 36, grna + grnb MO: n = 36. All bars represent mean ± s.e.m.

**Figure 2:**
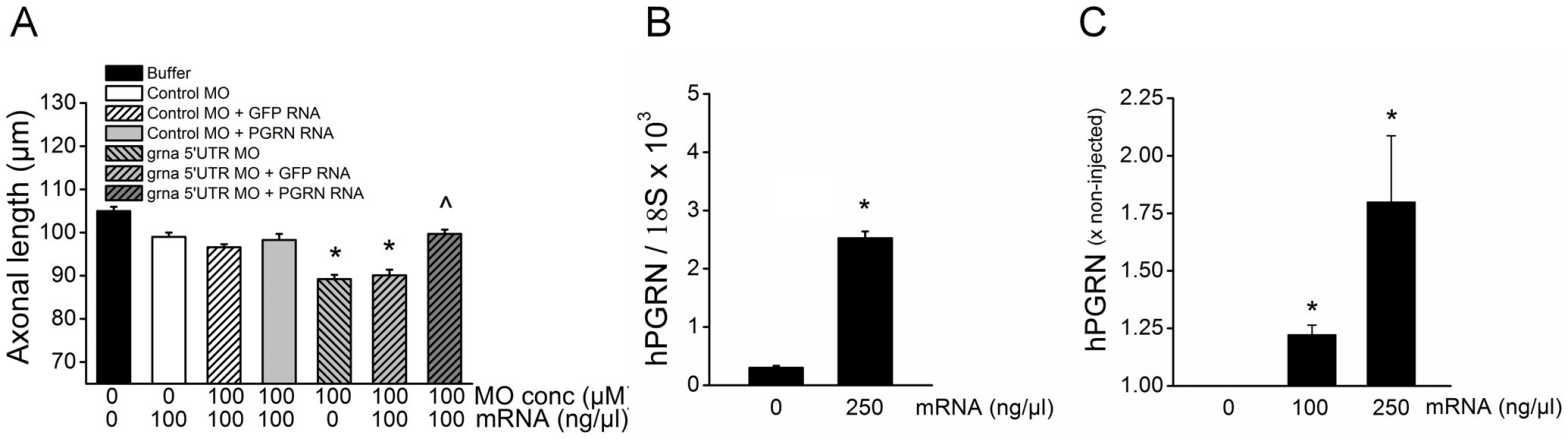
Overexpression of human PGRN mRNA prevents the decrease in axon outgrowth produced by knockdown of zebrafish PGRN. A) The decreased axonal length produced by knockdown of *grna* with a 5′UTR MO was rescued by co-expression of PGRN mRNA. * significantly different from grna CO MO, p<0.02; ^ significantly different from *grna* MO + GFP, p<0.0001. Bars represent mean ± s.e.m. Buffer injected: n = 20, CO-MO: n = 15, CO-MO + PGRN: n = 15, grna MO: n = 15, grna MO + GFP: n = 15, grna MO + PGRN: n = 13. B) Real time PCR analysis of cDNA reverse transcribed from RNA extracted from either non-injected or PGRN mRNA injected zebrafish embryos confirmed the presence of human PGRN mRNA in injected embryos (*p<0.0001). C) Quantification of human PGRN protein levels in non-injected and PGRN mRNA injected zebrafish embryos (24hpf) by ELISA confirmed the overexpression of human PGRN protein following PGRN mRNA injection (100 and 250 ng/µl). *Significantly different from non-injected, p<0.043 (post-hoc Wilcoxon Signed Ranks test) following a significant Friedman Test, p = 0.015.

Knockdown of *grna* and *grnb* separately with morpholino (MO) directed to either the start codon (ATG MO) or sequence within the 5′ untranslated region (5′ UTR morpholino) led to dose dependent decreases in axonal length (Figure 1A and B). The effect of knockdown of *grna* was more pronounced than that of knockdown of *grnb*. The *grna* and *grnb* MO together had a cumulative effect (Figure 1C). The axonal shortening induced by *grna* knockdown (using the 5′ UTR MO) was rescued by co-expression of human PGRN mRNA (Figure 2A), indicating that the effect was specifically caused by PGRN deficiency. Real time PCR, following reverse transcription of RNA extracted from 24 hours post fertilization (hpf) zebrafish embryos injected with PGRN mRNA (250ng/µl), confirmed the presence of human PGRN mRNA following injection (Figure 2B). Further, a human PGRN ELISA assay confirmed overexpression of human PGRN protein in zebrafish embryos injected with PGRN mRNA (Figure 2C).

### Mt-TDP induces a motor axonopathy

**Figure 3:**
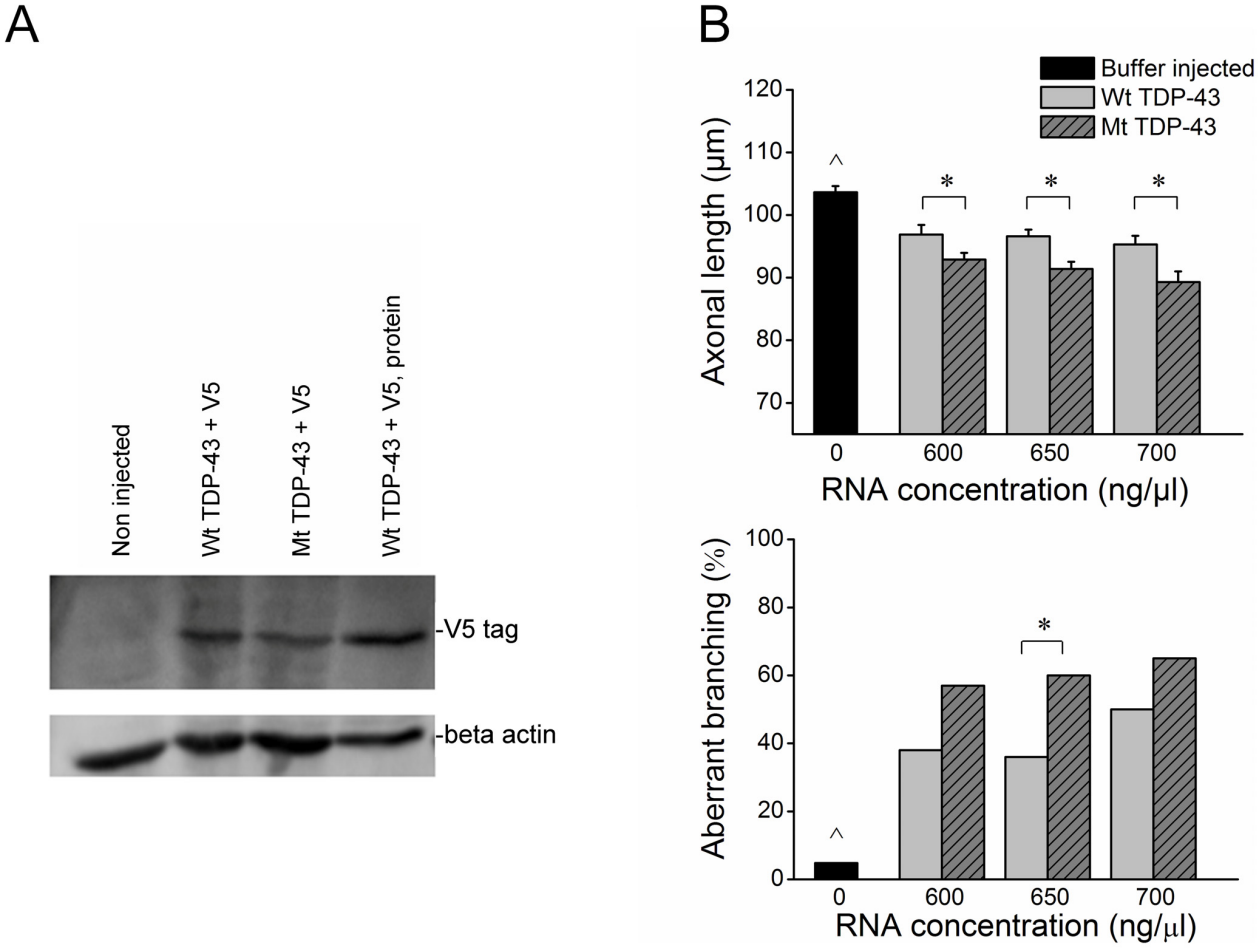
Overexpression of wild type (Wt) and mutant (Mt) TDP-43 mRNA produces motor axonopathies. A) Anti-V5 immunoblot confirmed similar expression levels of Wt and Mt TDP-43 following TDP-43 mRNA injection. Protein produced by *in vitro* translation of the Wt TDP-43 mRNA served as a positive control. B) Overexpression of Wt TDP-43 produced modest axonal shortening (∧p<0.001) and aberrant branching (∧p<0.001) in comparison with Buffer injection (n = 61). Mt TDP-43 expression (600 ng/µl: n = 37, 650 ng/µl: n = 64, 700 ng/µl: n = 26) produced a more pronounced effect, resulting in significantly shorter axonal lengths (*p<0.001) and more embryos affected by aberrant branching (*p<0.001) than Wt TDP-43 injected embryos (600 ng/µl: n = 35, 650 ng/µl: n = 59, 700 ng/µl: n = 45).

Injection of wild type (Wt) and mutant (Mt, A315T) TDP-43 mRNA resulted in overexpression of human TDP-43, as indicated by immunoblot of embryos 28 hours post injection (Figure 3A). Protein produced by *in vitro* translation of the Wt TDP-43 mRNA served as a positive control. Embryos overexpressing Wt TDP-43 exhibited modest axonal shortening and aberrant branching in comparison with buffer injected embryos (Figure 3B). Nevertheless, mutant TDP-43 expression produced a more pronounced decrease in axonal length compared with Wt TDP-43 expressing embryos (Figure 3B), despite the embryos having equal body lengths (p = 0.62). Overexpression of mutant TDP-43 also produced an outgrowth defect resulting in significantly more branching in the Mt TDP-43 than Wt TDP-43 expressing embryos when injected with 650 ng/µl mRNA. Signs of axonopathy (decreased axonal length and increased branching) were also evident when Wt TDP-43 was injected at higher concentrations (>650 ng/µl), decreasing the difference between Wt and Mt TDP-43 expressing embryos at these concentrations.

### Overexpression of human PGRN mRNA is protective against the mutant TDP-43 induced axonopathy

**Figure 4:**
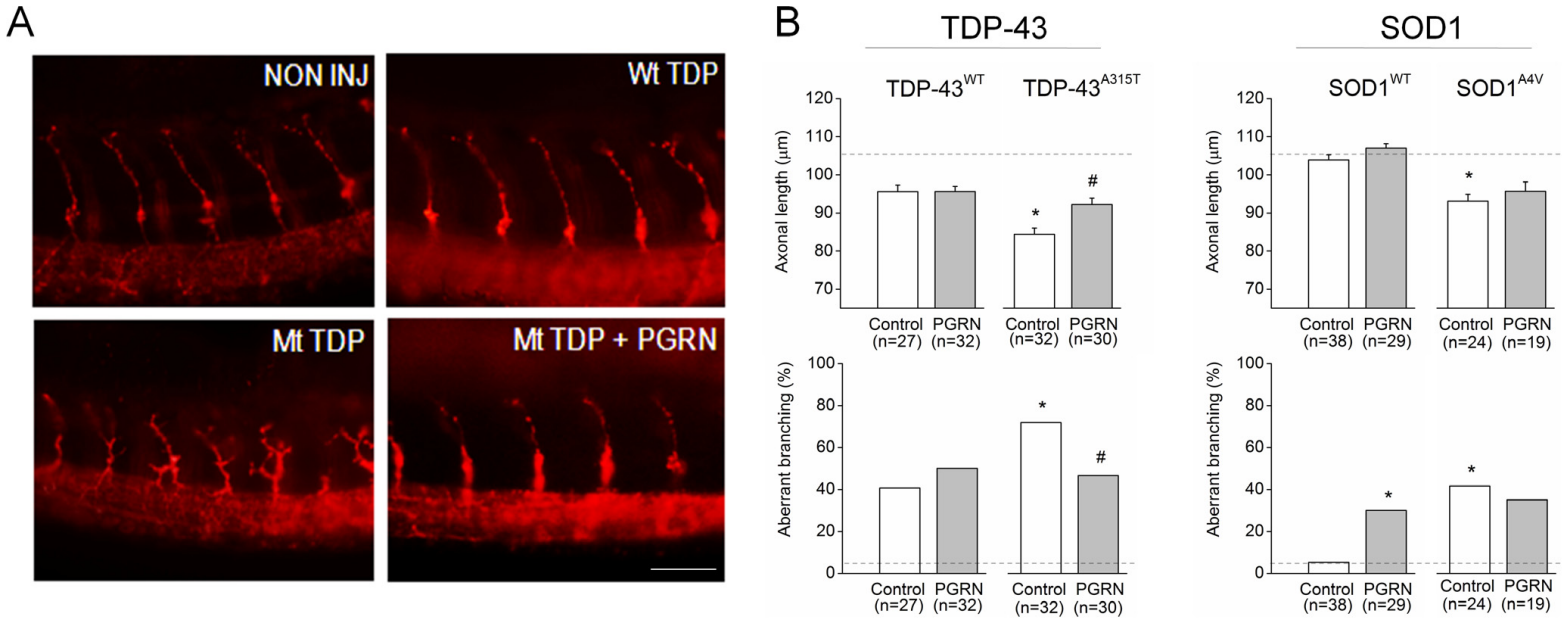
Co-expression of PGRN rescues the axonopathy induced by Mt TDP-43 but not Mt SOD1. A) Staining of primary motor axons with an anti-synaptic vesicle 2 revealed a decrease in axonal length and increase in aberrant branching in embryos expressing Mt TDP-43 compared with Wt TDP-43. These signs of axonopathy were reduced by co-expression of human PGRN. B) Zebrafish embryos co-expressing Mt TDP-43 and control mRNA (GFP) exhibited decreased axonal outgrowth and increased aberrant branching compared to embryos expressing Wt TDP and GFP (p<0.001 and p = 0.016, respectively). However, co-injection with the equivalent dosage of PGRN mRNA (250 ng/µl) rescued both axopathies described (p<0.043). Mt SOD1 produced motor axon shortening (p<0.001) and increased branching (p<0.001) in comparison with Wt SOD1, as described previously [25]. PGRN co-expression had no significant effect on the Mt SOD1 induced axonopathy but did increase aberrant branching in Wt SOD1 injected embryos (p = 0.006). ∧ indicates significantly different from buffer, * significantly different from ‘Wt + GFP’, and # significantly different from ‘Mt + GFP’. Bars represent mean ± s.e.m and the number of replicates per group is displayed below each bar.

To test for a neuroprotective effect of PGRN mRNA on the mutant TDP-43 phenotype, human PGRN mRNA was co-injected with Mt TDP-43 mRNA. Embryos co-injected with Mt TDP-43 and GFP mRNA, as a control for non-specific effects of RNA injection, exhibited similar axonal shortening and aberrant branching as embryos expressing Mt TDP-43 alone. Co-expression of PGRN mRNA with the Mt TDP-43 led to a significant increase in axonal outgrowth length, approaching that of Wt TDP-43 expressing embryos (Figure 4A and B). Co-expression of PGRN mRNA also decreased the amount of aberrant branching seen in Mt TDP-43 expressing embryos. Conversely, overexpression of the same amount of PGRN mRNA with Wt TDP-43 led to increased aberrant branching.

### Human PGRN mRNA is not protective against the axonopathy induced by mutant SOD1

Our laboratory has previously demonstrated that Mt SOD1 overexpression leads to motor axonopathies similar to that described here for Mt TDP-43 [25]. Co-expression of PGRN mRNA had no significant protective effect on the Mt SOD1 induced axonopathy (Figure 4B). Co-expression of PGRN with Wt SOD1 led to an increase in aberrant branching, similar to that seen when PGRN was co-expressed with Wt TDP-43.

### TDP-43 localization in zebrafish embryos

**Figure 5:**
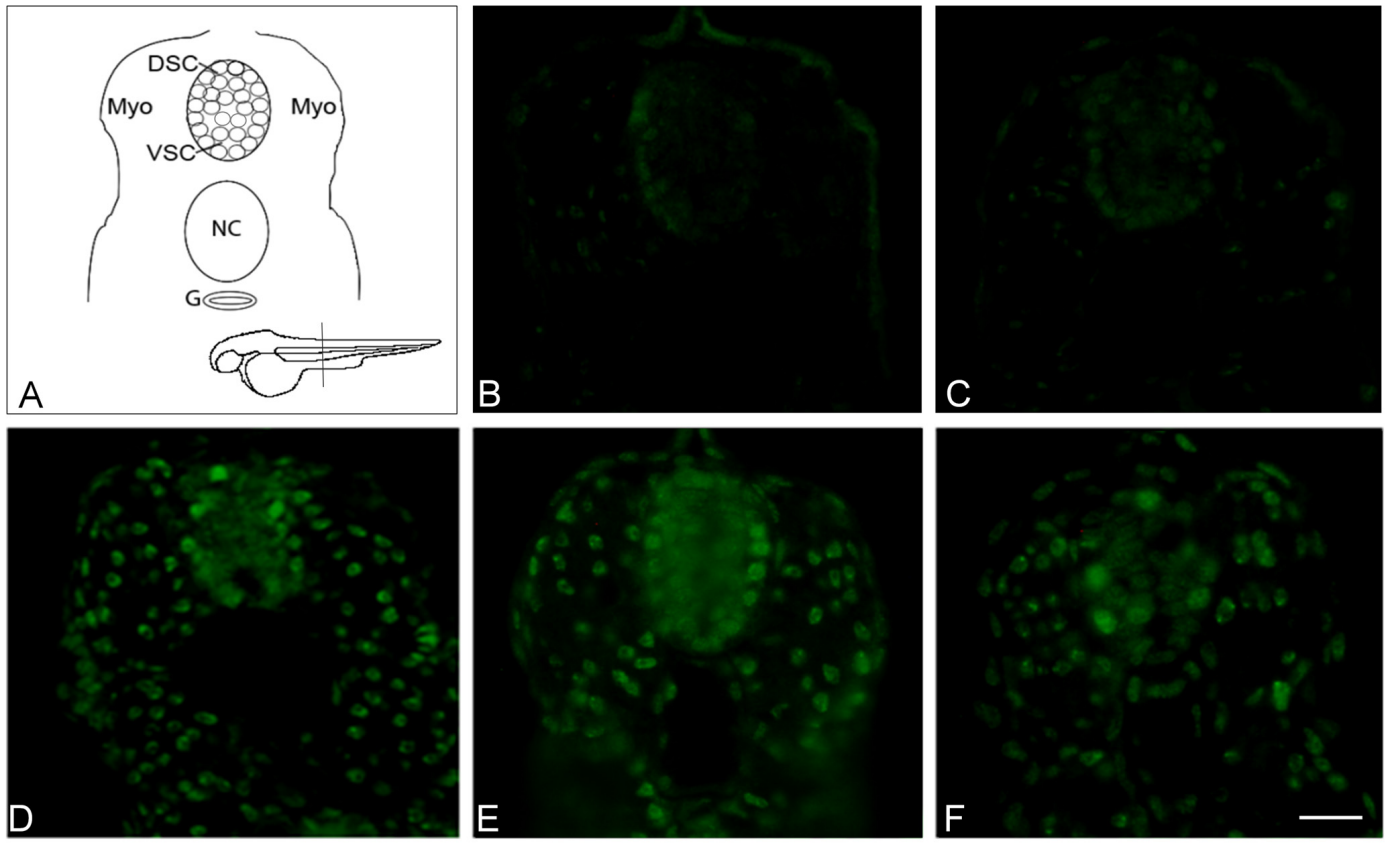
TDP-43 localization in zebrafish embryos. Immunofluorescent staining of endogenous zebrafish TDP-43 and overexpressed human TDP-43 was performed in transversely sectioned 30 hpf embryos in order to allow imaging of the spinal cord (shown in schematic diagram, A). TDP-43 localization was nuclear in all embryos examined (B: non injected, C: PGRN MO injected, D: Wt TDP-43 injected, E: Mt TDP-43 (A315T) injected, and F: co-expressing Mt TDP-43 and PGRN). The scale bar indicates a distance of 25 µm. Abbreviations: DSC, Dorsal spinal cord; VSC, Ventral spinal cord; Myo, myotomes; NC, notochord; G, gut.

Immunofluorescent staining of endogenous zebrafish TDP-43 and overexpressed human TDP-43 was performed in transversely cryosectioned 30hpf embryos. TDP-43 immunoreactivity was detected throughout the body of embryos, including in the ventral spinal cord and forebrain. A nuclear staining pattern was found in all embryos examined (non-injected, PGRN Morpholino injected, Wt TDP-43, Mt TDP-43 and Mt SOD1) (Figure 5). As expected, the intensity of TDP-43 immunofluorescence was greater in embryos overexpressing human TDP-43.

## Discussion

The data reported here demonstrate for the first time the neurotrophic action of PGRN *in vivo*. Decreased PGRN levels, produced by coinjected of morpholinos against zebrafish PGRN gene homologues, *grna* and *grnb*, resulted in a substantial decrease in axonal length. This decreased axonal outgrowth was produced by morpholinos against both homologues simultaneously, as well as separately. Further, use of morpholino acting in two different manners to prevent translation, one that binds to the start codon of the PGRN sequence and another that binds to a 5′ untranslated sequence, preventing the 40S ribosomal subunit from reaching the start codon, led to the same effect. Non-specific effects of morpholino injection were eliminated by comparison of all effects with the morphology of embryos injected with the same concentration of a control morpholino that contains the same sequence but with five base pairs changed so that they do not bind to the target sequence. The specificity of the morpholino effect was further clarified by a reversal of the decreased axonal outgrowth through co-injection of human PGRN mRNA.

Our studies confirm a previous report that expression of mutant TDP-43 induces a motor neuron axonopathy in zebrafish embryos [26]. We further report that in addition to mutant TDP-43, high levels of Wt TDP-43 also produce axon abnormalities. In larger models, such as the mouse, overexpression of Wt TDP-43 has been reported to result in neuronal loss, and in turn motor dysfunction [27]. These results raise the possibility that altered expression of Wt TDP-43 may also be disease causing in sporadic ALS patients. Further studies are needed to elucidate if raised Wt TDP-43 levels are present in sporadic ALS patients, perhaps due to variations in the non-coding region of the gene [28].

Co-expression of PGRN mRNA proved to be protective against the mutant TDP-43 induced axonopathy described. This co-expression increased axonal outgrowth length to near normal levels, and also decreased the degree of aberrant branching found in mutant TDP-43 expressing embryos. Interestingly, PGRN expression had no effect on the mutant SOD1 induced axonopathy and actually lead to a slight increase in branching within wild type SOD1 expressing embryos, wherein axonal length is at near maximal levels. The differential results for TDP-43 and SOD1 suggest that the pathogenic mechanisms through which TDP-43 and SOD1 lead to motor neuron degeneration may be different, not only in their molecular pathways and morphological hallmarks, but also in receptivity to protection by exogenous neurotrophic factors. This is consistent with previous reports that whilst TDP-43 pathology is present in the brains and spinal cords of a majority of ALS patients [12], [29], it is not present in patients with SOD1 mutations [23], [24].

We did not see abnormal TDP-43 sequestration following PGRN knock down or mutant TDP-43 overexpression, consistent with previous results in other animal models [26], [30], [31], [32]. The only effect of injection of TDP-43 mRNA on TDP-43 immunoreactivity was an increase in the intensity of the staining compared to non-injected and PGRN MO injected embryos. These data suggest that, at least in the zebrafish model, mutant TDP-43 expression and decreased PGRN levels can exert their harmful effects without TDP-43 mislocalization occurring.

Our results demonstrate that PGRN is required for normal axonal outgrowth in zebrafish embryos. We also report for the first time the neuroprotective effect of supplemental PGRN levels against mutant TDP-43 induced abnormalities. We thus suggest that PGRN can have neurotrophic and neuroprotective effects *in vivo* and that its therapeutic use for some forms of motor neuron degeneration should be further investigated.

## Methods

### Constructs, mRNA production and morpholino

Antisense morpholinos (MO) against the start codon (ATG) or 5′UTR of *grna* and *grnb* were designed and obtained from Gene Tools (Philomath, OR, USA). Control morpholinos (CO MO), comprised of the same sequence as their matching antisense MO except for five mismatched bases, were also obtained from Gene Tools. The sequences of the morpholino used are as follows:

grna ATG MO: CATTTTTTGAGCAGGTGGATTTGTG

grna ATG CO MO: CAATTTTTCAGCACGTGAATTTCTG

grnb ATG MO: AGGCTATGAAAGCTGCACGCACCAT

grnb ATG CO MO: AGCCTATCAAACCTGCACCCACGAT

grna 5′ UTR: TTTTTGAGCAGGTGGATTTGTGAAC

grna 5′ UTR CO MO: TATTTGACCACGTGCATTTGTCAAC

grnb 5′ UTR: ACAGATGAAAAGCCATGAACGACTT

grnb 5′ UTR CO MO: ACACATCAAAACCCATCAACGAGTT

cDNA of wild type (Wt) human TDP-43 was purchased from OpenBiosystems (Huntsville, AL) in the pCMVSport6.1 vector and subcloned into the pcDNA4/V5-HisA vector (Invitrogen). Mutagenesis was performed to produce the A315T mutation using the Stratagene QuikChange Site-Directed Mutagenesis Kit with the following primers:

A315T, Forward: GATGAACTTTGGTACGTTCAGCATTAATCC

A315T, Reverse: GGATTAATGCTGAACGTACCAAAGTTCATC

The construct was linearized by *Stu*I and mRNA was transcribed from the linear transcript using the mMESSAGE mMACHINE T7 Kit (Ambion, Huntingdon, UK) followed by purification with a MEGAclear™Kit (Ambion, Huntingdon, UK). mRNA-concentration was determined using a NanoDrop 1000 spectrophotometer (Thermo Scientific, Waltham, MA). The progranulin mRNA was similarly produced from a Topo TA Cloning vector (Invitrogen), containing the full human PGRN coding sequence, following linearization with *Hin*dIII.

### Zebrafish maintenance and injection

All experiments were approved by, and performed in accordance with the guidelines of, the Ethical Committee for Animal Experimentation, K.U. Leuven (project approval number P021/2010). Adult zebrafish (AB strain) and embryos were maintained under standard laboratory conditions. Zebrafish embryo microinjections were made using a FemtoJet injection setup (Eppendorf, Hamburg, Germany). Each injection was made in the 1–4 cell stage of the zebrafish embryo and involved delivery of 2.14 nl of mRNA/MO solution, accomplished by an injection pressure of less than 4.5 psi, which produced a droplet diameter of 160 µm on a micrometer. Embryos were then stored in E3 solution (5 mM NaCl, 0.17 mM KCl, 0.33 mM CaCl2 and 0.33 mM MgSO4 and 0.1% Methylene Blue) at 27.5–28.5°C.

### Analysis of motor neuron outgrowth

At 30 hours post-fertilization (hpf), morphologically normal zebrafish embryos were fixed in 4% paraformaldehyde in phosphate-buffered saline (PBS) and immunostained using mouse anti-synaptic vesicle 2 (1/200; Developmental Studies Hybridoma Bank, University of Iowa, Iowa City, IO, USA) and secondary Alexa Fluor 555 anti-mouse antibody (1/500; Molecular Probes, Eugene, OR, USA) in order to visualize motor neurons. Observers blind to injection and treatment conditions measured the axonal length of the first five ventral motor axons after the yolk sac in each embryo using Lucia software (version 4.9) and the average of these five lengths was calculated for each embryo. Motor axons were scored as affected by aberrant branching when two or more axons per embryo branched at, or medial to, the ventral edge of the notochord (all axons on each side of the embryo were checked).

### Real time PCR

A real time PCR was used to quantify the amount of human progranulin mRNA delivered via injection into zebrafish embryos. RNA was extracted from zebrafish embryos (50 embryos per group, with three replicates per condition), using Trizol (Invitrogen, San Diego, CA) and isopropanol purification. cDNA was obtained from the extracted RNA by reverse transcriptase PCR (QuantiTect reverse transcription kit). The amount of human proganulin DNA was quantified by real time PCR with the following components:

Forward primer: 5′ CGG AGG AGC CAG CTA CAG 3′

Reverse primer: GAT5′ GCC TGC TCA GTG TTG TG 3′

and probe: FAM-CCC CTT CTG GAC AAA TG-NFQ

Thermal cycling was performed on a 7300 Sequence Detection System (Applied Biosystems, Carlsbad, CA). All samples were normalized to the level of 18S RNA.

### Evidence of protein expression

Zebrafish embryos were dechorionated at 24–28 hpf and transferred to a solution of E3 solution containing 1 mM PMSF. The yolksac of the embryo was removed by suction through a narrow glass pipette three times. The deyolked embryos were then transferred to eppendorf tubes where the supernatant was removed and 0.75 µl/embryo T-PER lysis buffer (Peirce, Rockford, IL, USA) containing one tablet of Complete Protease Inhibitors (Roche, Mannheim, Germany) per 25 ml and Phenylmethylsulfonyl fluoride (PMSF, final concentration 1 mM), was added to lyse the sample. The samples were sonicated for 20 sec and protein concentration was determined using the Micro-BSA Protein Assay Reaction Kit (Pierce). For the TDP-43 immunoblot, 100 µg of zebrafish protein lysate and 5 µg of human Wt TDP-43 protein, produced by *in vitro* translation of the Wt TDP-43 mRNA using a Retic Lysate IVT™ kit (Ambion, Huntingdon, UK), was separated on a SDS page gel and processed as described previously [33]. Protein bound to the PVDF membrane was probed with an antibody against the V5 tag (Invitrogen) that is fused to TDP-43 in the injected mRNA, followed by anti-mouse HRP-coupled secondary antibody (1∶5000; Santa Cruz, Santa Cruz, CA, USA). The protein bands were visualized by chemiluminescence (ECL Western blot substrate, Peirce) and scanned on a Biospectrum AC imaging system (UVP, CA).

Human PGRN protein levels were measured by sandwich ELISA on protein lysates obtained in the same manner as described above for immunoblot analysis. Multiple protein lysate samples were prepared for each condition (non-injected: n = 6, 100 ng/µl PGRN mRNA injected: n = 6, and 250 ng/µl PGRN mRNA injected: n = 5), with each sample containing approximately forty embryos. For the ELISA assay, 96-well plates were coated overnight at 4°C with 2 µg/ml anti-human monoclonal PGRN antibody (R&D Systems) in phosphate-buffered saline (PBS). After blocking with 2% Bovine Serum Albumin, protein lysates diluted in PBS/0.004% Tween 20/0.1% BSA (or PBS alone) were incubated at room temperature for 2 hours. All wells were then incubated with anti-human biotinylated PGRN antibody (R&D systems), followed by avidin/biotin horseradish peroxidase complex (Vector Laboratories, Burlingame, CA, diluted 1∶100). The chromogenic reagent o-phenylenediamine (OPD) was applied to all wells of the plate (30 min, room temperature) and the resulting color reaction was stopped with 4 M H_2_SO_4._ The plate was read on a VictorX3 plate reader (Perkin Elmer, Waltham, MA) at an absorbance of 490 nm. The resultant progranulin protein concentrations were normalized to the total protein content of the sample, and graphed normalized to the amount of background signal (the human progranulin concentration of the non-injected sample).

### TDP-43 immunofluorescence in zebrafish sections

To allow immunofluorescent staining of sectioned zebrafish embryos 30hpf embryos were fixed in 4% paraformaldehyde overnight, followed by overnight cryoprotection incubation in 20% sucrose. Embryos were then mounted in Tissue-Tek OCT compound (Sakura Finetek, Zoeterwoude, The Netherlands), frozen and transversely cryosectioned at a thickness of 20 µm. Antigen retrieval was performed by boiling sections in sodium citrate buffer (10 mM sodium citrate, 0.05% Tween 20, pH 6.0) for 30 min. Non-specific staining was blocked by incubation of sections in blocking solution (PBS containing 5% normal horse serum, 5% bovine serum albumin and 0.1% Triton X-100) for 1 hr. Sections were then incubated in primary antibody (1/200, polyclonal rabbit anti-TDP-43, ProteinTech Group Inc., Chicago, IL) diluted in PBS with 0.1% Triton X-100. Incubation with a fluorescent secondary antibody (anti-rabbit Alexa Fluor 488 antibody, 1/500; Molecular Probes, Eugene, OR, USA) followed.

### Statistical analysis

Statistics were performed using SPSS 16.0. Axonal lengths were analyzed with one-way ANOVA followed by a Bonferroni post-hoc test. For comparison of the rate of aberrant branching (percentage data) chi-squared tests were used. Real time PCR data was also analyzed with one-way ANOVA. The human PGRN protein concentration data obtained from an ELISA assay was analyzed with a non-parametric Friedman repeated measures test, followed by a post-hoc Wilcoxon signed ranks test. All P-values <0.05 were taken as significant.
